# The decreased of peripheral blood natural killer cell is associated with serum IL-2 level in the renal tubular acidosis in patients with primary sjogren’s syndrome

**DOI:** 10.1186/s12865-023-00550-7

**Published:** 2023-06-30

**Authors:** Liyun Cheng, Lu Liu, Ronghui Su, Huanhuan Yan, Xiaoyu Zi, Chong Gao, Xiaofeng Li, Caihong Wang

**Affiliations:** 1 Department of Rheumatology, The Second Hospital of Shanxi Medical University, Taiyuan, Shanxi China; 2 Pathology, Joint Program in Transfusion Medicine, Children’s Hospital, Brigham and Women’s Hospital, Harvard Medical School, Boston, MA USA

**Keywords:** Primary Sjogren’s Syndrome, Renal tubular acidosis, Interleukin 2, Natural killer cells, Regulatory T cells

## Abstract

**Background:**

Primary Sjogren’s Syndrome (pSS) is a lymphoproliferative disease with autoimmune characteristics, which is characterized by lymphocyte infiltration of exocrine glands and involvement and dysfunction of extraglandular organs. Renal tubular acidosis (RTA) is a common renal involvement in pSS. This study investigated the phenotypic characteristics of peripheral blood lymphocyte subsets and cytokines in pSS patients complicated with RTA (pSS-RTA).

**Method:**

This retrospective study included 25 pSS patients complicated with RTA and 54 pSS patients without RTA (pSS-no-RTA). To examine the level of peripheral lymphocytes subsets, flow cytometry analysis was used. The level of serum cytokines were detected by flow cytometry bead array(CBA). The influencing factors related to the occurrence of pSS-RTA were identified through logistic regression analyze.

**Results:**

The absolute number of CD4 + T cells and Th2 cells in peripheral blood were decreased in pSS-RTA patients than pSS-no-RTA patients. Moreover, the absolute number of NK cells and Treg cells were also decreased in pSS-RTA patients than pSS-no-RTA. The level of serum IL-2 was higher in pSS-RTA patients than pSS-no-RTA patients, and is negatively correlated with the number of NK cells, the number and percentage of Th17 cells, and Th17/Treg. Serum IL-2 level is also correlated with various cytokines. Multivariate logistic analysis proved that elevated ESR and ALP were risk factors for pSS complicated with RTA, while Treg was a protective factor.

**Conclusion:**

The increase of serum IL-2 level and the decrease of peripheral blood NK cells and Treg cells may be the immune mechanism of the development of pSS-RTA disease.

## Introduction

Primary Sjogren’s Syndrome (pSS) is a chronic inflammatory autoimmune disease characterized by lymphocyte infiltration of the exocrine glands, such as lacrimal gland and salivary gland [1]. As the second most common chronic autoimmune rheumatological condition, it usually occurs in women between 40 and 50 years old. In addition to the damage of lacrimal and salivary glands and the decline of function, multiple non-exocrine organ systems may also be implicated, such as the kidney [2, 3]. Tubulointerstitial nephritis (TIN), the most common pathogenic form of kidney injury in pSS, is characterized by inflammation of mononuclear lymphocytes in the interstitium[4], which is mostly manifested as renal tubular acidosis[5]. Long-term RTA can cause aberrant bone metabolism, urinary calculi, osteoporosis, and osteomalacia, all of which affect the quality of life for patients. Because the clinical symptoms are usually insidious, even prior to dry symptoms, the diagnosis of pSS combined with nephropathy is facing challenges. Therefore, greater research on the risk factors and pathological basis of pSS-RTA is required in order to develop more potent methods for early detection and intervention.

The pathogenesis of pSS has not been fully clarified so far. In the last decade, efforts have been made to describe the role of various T cell populations in pSS. Quantitative and qualitative changes in immune cell subsets have been shown in pSS, including T and B lymphocyte numbers and functions [6]. Research shows the decrease of CD4 + T cells and the increase of B cells in pSS patient, when compared with health [7]. In addition, studies have shown that the number of T helper cell 17 (Th17) cells in the peripheral blood of pSS patients is reduced compared to healthy subjects[8].However, the role of regulatory T (Treg) cells in pSS remains controversial. Study shows that Treg cells were decreased in peripheral blood of patients with pSS[9]. But another study indicates Treg cells shows no change between pSS patients and health [10]. Innate immune cells, particularly natural killer (NK) cells, are also heavily engaged in the pathogenesis of pSS in addition to helper T cells. For instance, it has been suggested that NK cells that express NKp30 interact with epithelial cells to exacerbate the inflammatory state in the salivary gland by secreting interferon-γ (IFN-γ)[11]. NK cells exert immune function by recognizing and clearing transformed and virus infected autologous cells, and decline in pSS[12].

At present, it is generally believed that humoral and cellular immunity are the causes of distal tubular injury in patients with autoimmune diseases [13]. pSS damages the kidney and causes various clinical symptoms through the deposition of immune complexes or infiltration of renal tubular lymphocytes. The histological findings of pSS-RTA are frequently described as a diffuse lymphocytic infiltration of renal interstitial tissue with plasma cells, implying that pSS in combination with RTA is connected with organismal immune function[14]. However, few studies have explored the role of innate immune indicators in the identification of pSS-RTA patients. Interleukin 2 (IL-2) is an important cytokine with multiple effects: it can not only act as an inflammatory factor to promote the proliferation of T cells, enhance the ability of NK cells, and enhance the anti-tumor immune response of the body [15–17], but also promote the proliferation of Treg cells to control inflammatory response and maintain immune tolerance. In patients with pSS-RTA, the relationship between serum IL-2 level and disease activity, Th17/Treg immune balance, peripheral blood lymphocyte subsets and other serum cytokines has rarely been reported.

The purpose of this project is to detect the level of peripheral blood immune cells in patients with pSS-RTA, clarify the immune cells that may participate in the occurrence of the disease and the differences of related peripheral blood immune indexes, and to investigate the relationship between serum IL-2 level and lymphocytes, and other cytokines in pSS-RTA patients.

## Materials and methods

### Clinical data

These 79 pSS patients (2 males and 77 females) were admitted to the Rheumatology and Immunology Department of the Second Hospital of Shanxi Medical University from May 2017 to October 2021. All patients included in this study met the 2002 American–European Consensus Group criteria[18] or 2016 American College of Rheumatology/European League Against Rheumatism SS classification criteria[19]. pSS-RTA patients met the diagnostic standard of RTA: Hyperchloremic metabolic acidosis, serum potassium < 3.5mmol/l, urine pH > 5.5. Thirty-three healthy controls of similar age were recruited from the physical examination center. The pSS patients included pSS-RTA group (N = 25) and pSS-no-RTA group(N = 54). The 33 healthy controls (HCs) included in this study had no evidence of autoimmune diseases, acute or chronic infectious diseases and no history of cancer. The data of all patients include demographic characteristics, clinical manifestations and the following laboratory data: including blood cells, erythrocyte sedimentation rate (ESR), C-reactive protein (CRP), renal function, electrolytes, Rheumatoid factor (RF), anti-SSA antibody, anti-SSB antibody, anti-nuclear antibodies (ANA), anti-ENA antibody, anti-Ro-52 antibody, complement 3(C3) and complement 4(C4), Immunoglobulin A(IgA), Immunoglobulin G(IgG), Immunoglobulin M(IgM), and the number and percentage of peripheral blood lymphocyte subsets. All blood samples used to test laboratory data were collected on an empty stomach in the morning. The study was approved by the Second Hospital of Shanxi Medical University ethics committee (Approval (2019) KY No. (105)), and all participants signed written informed consent.

### Detection of lymphocytes by flow cytometry

The venous blood was collected in a heparin anticoagulant tube, and peripheral blood mononuclear cells were isolated by density gradient centrifugation (Ficoll-hypaque). Adjust the monocytes to the appropriate concentration, and then use the corresponding antibodies to take the incubated cells.

For analysis of T lymphocytes, B lymphocytes and NK cells population, cells were incubated with surface markers antibodies, anti-CD3-FITC, anti-CD4-APC, anti-CD8-PE, anti-CD45-PerpCP, CD16 + CD56-PE, and CD19-APC. Cell types were defined as CD3 + CD19- total T cells, CD3-CD19 + B cells, CD3 + CD4 + T cells, CD3 + CD8 + T cells, and CD3-CD16 + CD56 + NK cells.

**Fig. 1:**
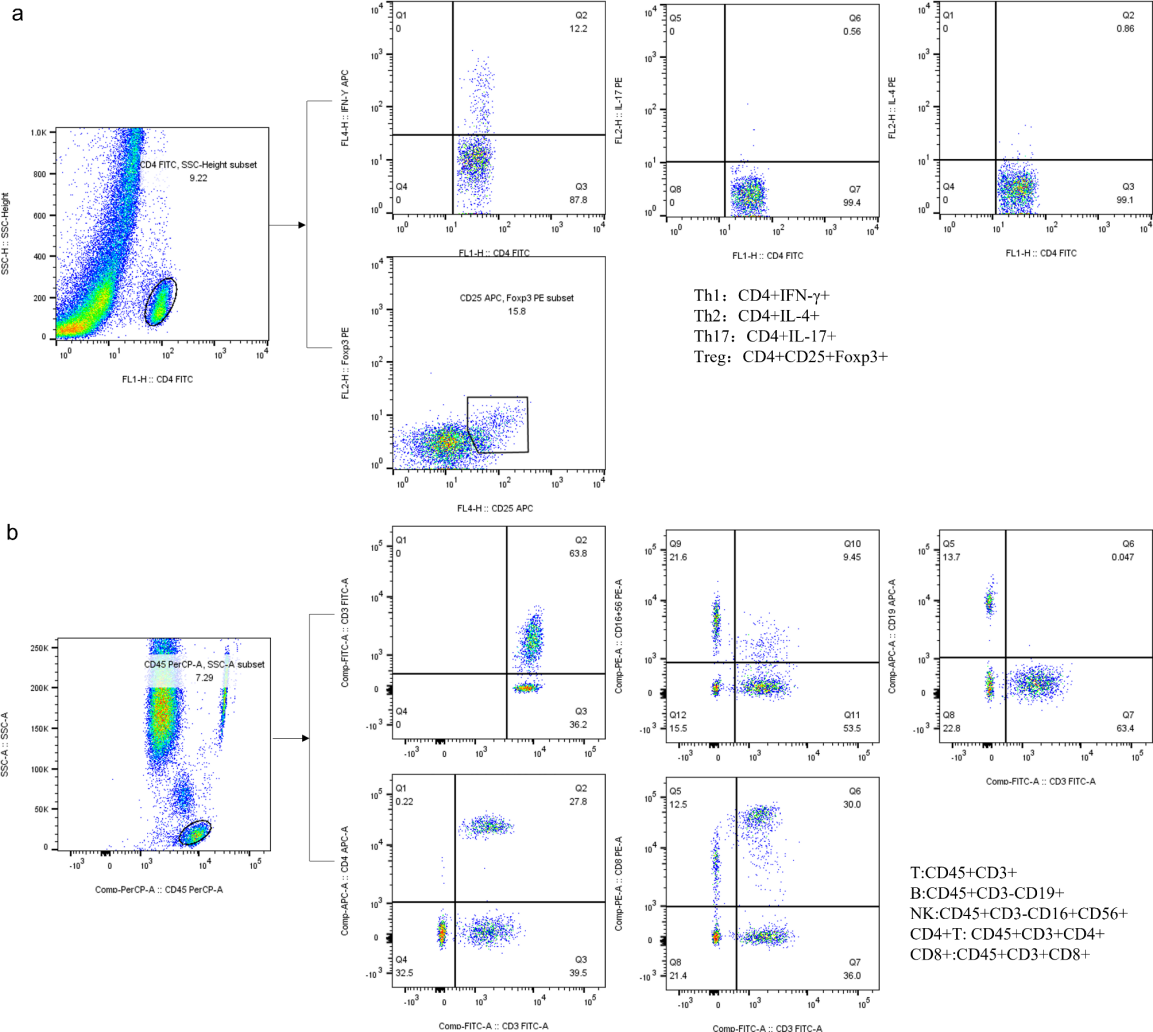
Representative pictures of flow cytometry. T cell: CD45 + CD3+; B cell: CD45 + CD3- CD19+; NK cell: CD45 + CD3- CD16 + CD56+; CD4 + T cell: CD45 + CD3 + CD4+; CD8 + T cell: CD45 + CD3 + CD8+; Th1 cell:CD4 + INF-γ+; Th2 cell: CD4 + IL-4+; Th17 cell: CD4 + IL-17+; Treg cell: CD4 + CD25 + Foxp3+

Additionally, for analysis of CD4 + T cells subsets, T helper cell 1 (Th1) cells was labeled with anti-CD4-FITC and anti-IFN-γ-APC, T helper cell 2 (Th2) cells were labeled with anti-CD4-FITC and anti-IL-4-PE. anti-CD4-FITC and anti-IL-17 A-PE were used for detecting Th17 cells. anti-CD4-FITC, anti-CD25-APC, and anti-FoxP3-PE were used for detecting Treg cells. The principle of flow gate setting is shown in Fig. 1. The immunofluorescent antibodies used in this study were purchased from BD Biosciences, and the corresponding experiments were carried out according to the manufacturer’s recommendations (BD Biosciences, Franklin Lakes, NJ, USA). The percentage and absolute number of CD4 + T lymphocyte subsets were automatically calculated by BD multitest software (BD Biosciences).

### Cytokine levels detected by flow cytometry bead array

Within 1 h of sampling, the serum was isolated from venous blood within 1 h of sampling and stored at -20 °C. Flow cytometry was used to detect the expression of IL-2, Interleukin 4 (IL-4), Interleukin 6 (IL-6), Interleukin 10 (IL-10), Interleukin 17(IL-17), IFN-γ, and tumor necrosis factor-α (TNF-α). Jiangxi cellgene Biotechnology Co. Ltd. (Jiangxi, China) provided a CBA kit, which was utilized according to the manufacturer’s instructions; the results were expressed as pg/ml.

### Statistical analysis

Data without normal distribution were described by the median (interquartile rage, IQR). The Mann-Whitney U test was used to compare the difference. Data with normally distributed was expressed as mean ± standard deviation. Differences between variables were analyzed using independent sample t-test. When the data was categorical variable, we summarized this variable with frequency and compared differences using chi-square test or Fisher’s exact test. Spearman correlation analysis was used to illustrate the correlation between lymphocyte subsets and inflammatory indexes. To analyze the potential risk factors associated pSS-RTA patients, logistic regression was used. P-value was set at 0.05 for all hypothesis tests. All calculations were performed using SPSS 23.0 and GraphPad Prism software 8.0.

## Result

### Essential information

A total of 79 pSS patients were enrolled in our study, including 25 pSS-RTA patients and 54 pSS-no-RTA patients. Demographic information, clinical characteristics, and laboratory findings of all patients are presented in Table 1. The difference of age between the two groups was not statistically significant. The disease duration of the two groups was 59 and 60 months, respectively. There was no variance between the two groups in terms of some common clinical manifestations of pSS patients such as dry mouth, dry eye, and the incidence of osteoporosis the difference doesn’t make sense. Especially, the prevalence of nephrolithiasis in pSS-RTA patients was 26.08%, while no cases of nephrolithiasis were found in pSS-no-RTA patients. In terms of autoantibodies, the positive rate of increased RF was substantially greater in pSS-RTA patients than in pSS-no-RTA patients. The positive rate of ANA and anti-SSA or anti-SSB antibodies were not significantly different between the two groups, however the positive rate of anti-SSA antibody was higher than anti-SSB antibodies in both groups (Table 1).

In terms of laboratory examination, compared to pSS-RTA cases, the percentage of increased ALP in pSS-RTA patients was 36.0%, higher than the 11.11% in pSS-no-RTA patients. The mean level of estimated glomerular filtration rate (eGFR) in pSS-RTA group was (88.16 ± 21.01) ml/min/1.73m^2^, and that in pSS-no-RTA group was (104.84 ± 11.77), the renal function of the two groups was significantly different from each other. A comparison of immune-related indicators revealed that pSS-RTA patients had considerably higher percentage of ESR and IgA than pSS-no-RTA patients. However, there was no significant difference in IgM, IgG, C3, C4 and CRP between the two groups (Table 1).

### Peripheral blood Th17 cells increased and treg decreased in pSS patients

**Fig. 2:**
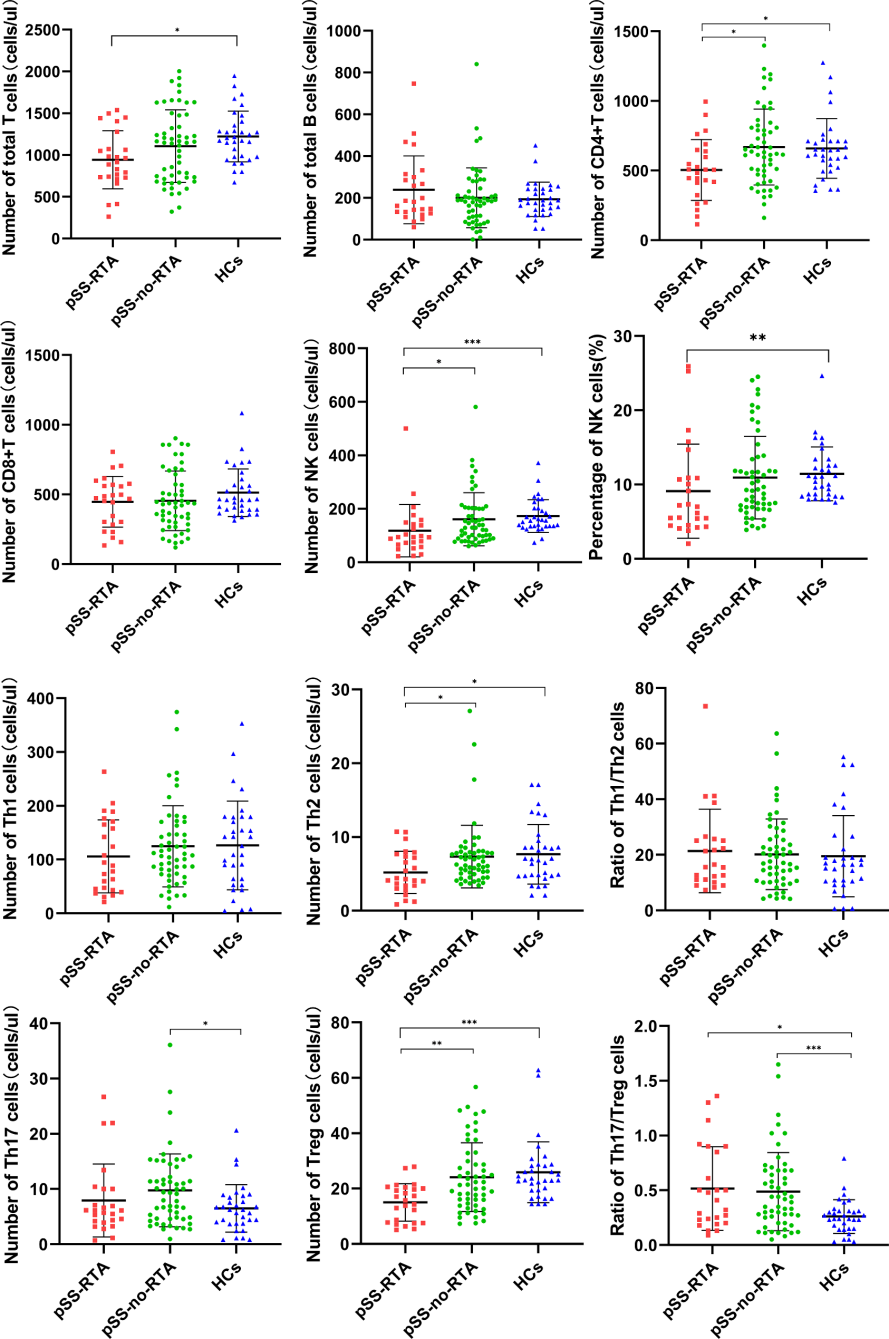
The level of peripheral lymphocyte and CD4 + T lymphocyte subsets in pSS-RTA, pSS-no-RTA and HCs. pSS: primary Sjogren’s syndrome; RTA: renal tubular acidosis; pSS-no-RTA: pSS without RTA; pSS-RTA: pSS with RTA. HCs: healthy controls; *P < 0.05, **P < 0.01, ***P < 0.001

To assess the immune function of all patients, we analyzed the numbers and proportions of various lymphocyte subsets in 79 pSS patients and 33 healthy controls. The absolute number of total T cells were decreased in pSS patients relative to healthy controls. Moreover, when compared with HCs, both the percentage and absolute number of NK cells were lower in pSS groups. Importantly, the absolute number of Treg cells in pSS patients were considerably lower than in HCs, with no difference in percentage of Treg cells. Moreover, the ratio of Th17/Treg cells was extremely higher pSS patients than HCs. Furthermore, when compared to HCs, pSS patients had a considerably higher percentage and absolute number of Th17 cells than the HCs (Table 2). Separately, the pSS-no-RTA groups had a larger percentage and absolute number of Th17 cells than the HCs, only the percentage of Th17 cells was higher in pSS-RTA than HCs, with no difference in the absolute number. Compared with the HCs, the absolute number of CD4 + T cells, Th2 cells and Treg cells in pSS-RTA patients decreased, and the ratio of Th17/Treg in pSS-RTA and pSS-no-RTA groups was higher (Table 3; Fig. 2).

### Peripheral NK cells and treg cells were decreased in pSS-RTA patients

When comparing the differences of peripheral lymphocyte subsets between the pSS-RTA patients and pSS-no-RTA patients, the results showed that pSS-RTA patients had lower absolute number of CD4 + T cells and NK cells(p=0.04997) than pSS-no-RTA patients. The differences in CD4 + T cell subsets between pSS-RTA and pSS-no-RTA patients were further analyzed. The result showed that pSS-RTA patients had lower number of Th2 cells than pSS-no-RTA patients. No obvious difference was indicated between the two groups when compared the number of Th1 cells. It is rather remarkable that the absolute number of Treg cells were significantly decreased in pSS-RTA group than pSS-no-RTA group. We are surprised that no significant difference was showed in the percentage and absolute number of Th17 between pSS-RTA and pSS-no-RTA. The result means that it is the decrease in Treg cells rather than the increase in Th17 is related to the pathogenesis of pSS-RTA. The level of total T lymphocytes, B lymphocytes and CD8 + T cells in pSS-RTA group and pSS-no-RTA group did not differ with each other (Table 3; Fig. 2).

### pSS-RTA patients had significantly greater levels of many cytokines than pSS-no-RTA patients

**Fig. 3:**
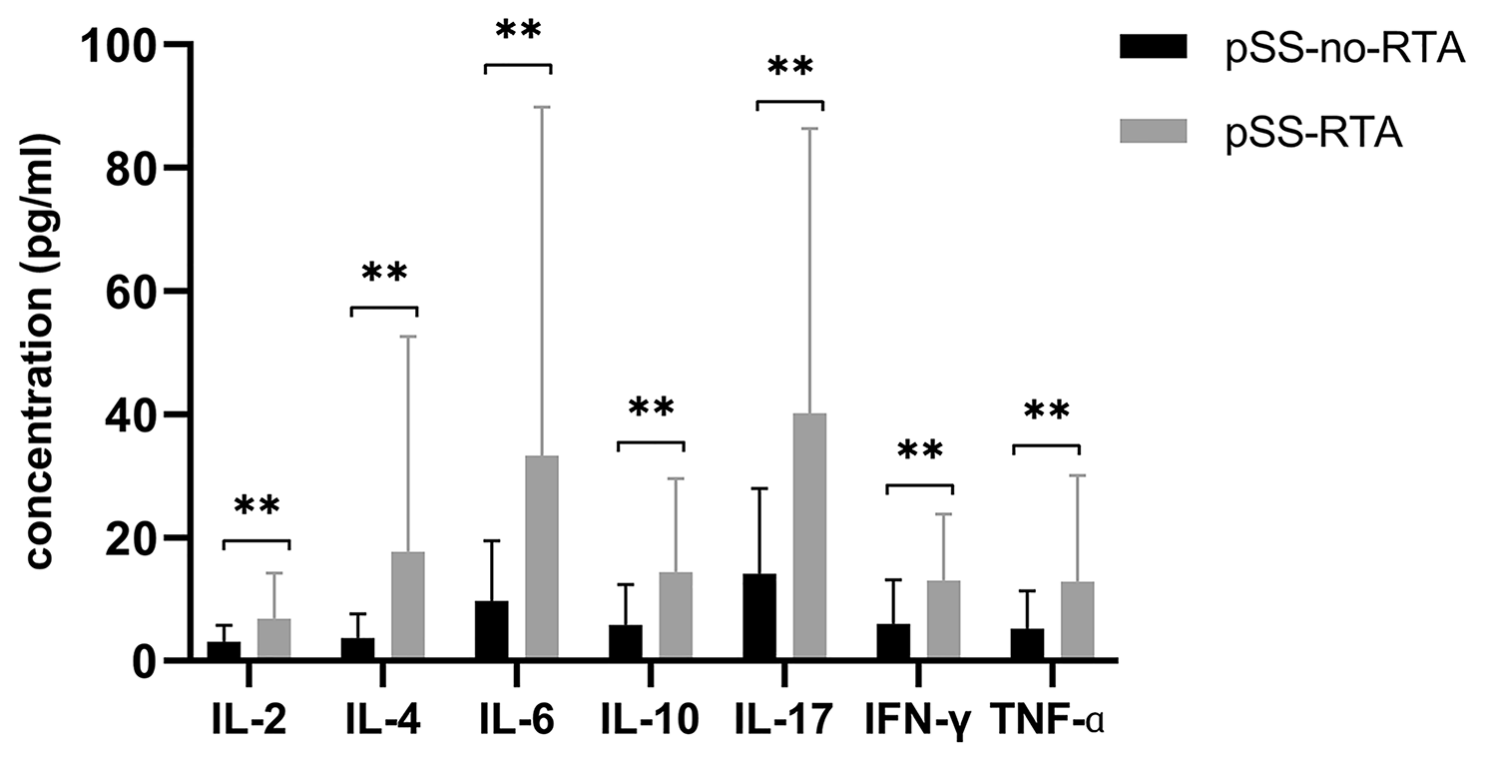
Cytokine levels (pg/ml) in pSS-RTA group(n = 17)and pSS-no-RTA (n = 52). IL-2: interleukin-2; IL-4: interleukin-4; IL-6: interleukin-6; IL-10: interleukin-10; IL-17: interleukin-17; INF-γ: interferon-γ; TNF-α: tumor necrosis factor-α. *P < 0.05, **P < 0.01

Regarding differences in cytokine levels between the pSS-RTA patients and pSS-no-RTA patients. The former group showed significantly higher levels of some cytokines, including IL-2 (3.18 vs. 6.89, p < 0.01), IL-4 (3.69 vs. 19.40, p < 0.01), IL-6 (11.72 vs. 42.31, p < 0.01), IL-10 (5.94 vs. 15.05, p < 0.01), IL-17(15.62 vs. 38.60, p < 0.01), IFN-γ (6.31 vs. 12.70, p < 0.01), TNF-α (5.37 vs. 13.29, p < 0.01) (Fig. 3). These results indicate that abnormalities in many cytokines are implicated in the pathogenesis of pSS-RTA.

### IL-2 level is correlated with various cytokines and lymphocytes

**Fig. 4:**
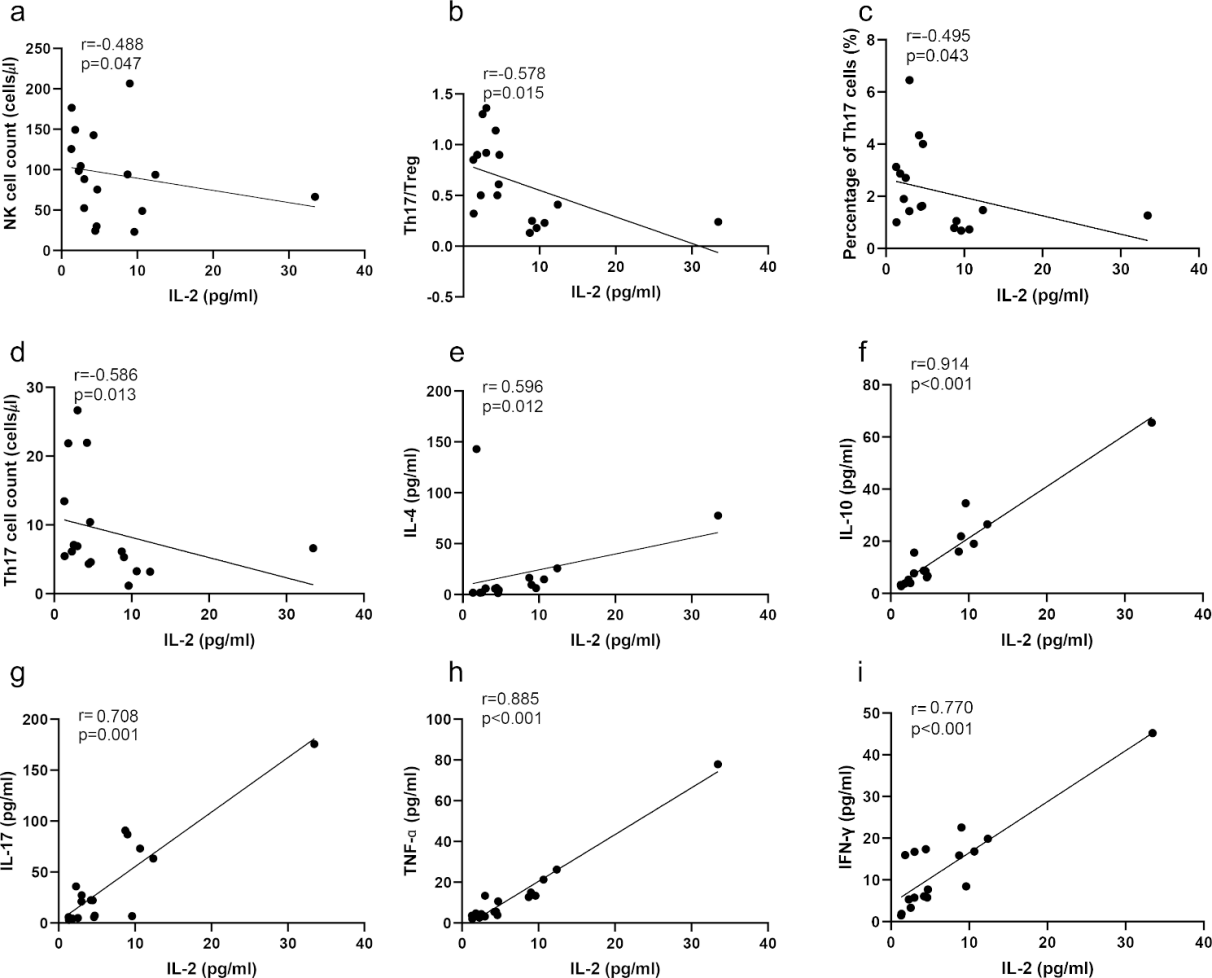
Correlation analysis of IL-2 with lymphocytes and cytokines. Correlation analysis between serum IL-2 level of pSS-RTA patients and lymphocyte number (a–d). Correlation analysis between serum IL-2 level of RA patients and IL-4, IL-10, IL-17, INF-γ, and TNF-α(e-i)

When we analyzed the correlation between IL-2 and lymphocyte subsets and other cytokines, we found that the level of IL-2 in serum of patients with pSS-RTA was negatively correlated with the absolute number of NK cells(r=-0.488, p = 0.047), Th17/Treg ratio (r=-0.578, p = 0.015), and the number and percentage of Th17 cells (r=-0.586, p = 0.013; r=-0.495, p = 0.043). When comparing the correlation between IL-2 level and other cytokines, it was found that IL-2 level in serum of patients with pSS-RTA was positively related to IL-4 (r = 0.596, p = 0.012), IL-10 (r = 0.914, p < 0.001), IL-17 (r = 0.708, p = 0.001) and IFN- γ (r = 0.77, p < 0.001) and TNF-ɑ (r = 0.885, p < 0.001). ESR and CRP are usually used to evaluate disease activity, and cytokines have no correlation with these indexes (Fig. 4).

### The decline of treg cell was related to the presence of RTA in pSS in multivariable models

Logistic regression analysis was applied to assess factors related to the presence of RTA in pSS. The results of univariate logistic analysis showed that the increase in Alkaline Phosphatase (ALP), ESR and IgA, as well as the positive RF were significant correlation with RTA in pSS. The absolute number of Treg cells and Th2 cells were also found to be associated with RTA in pSS. Further research incorporated these factors into multivariate analysis. Importantly, multivariate logistic analysis revealed that the increase in ESR and ALP were the risk factors for pSS complicated with RTA, while Treg was the protective factor (Table 4).

## Discussion

The onset of pSS-RTA is insidious, and its clinical manifestations are diverse. Therefore, clinicians need to have a more comprehensive understanding of the risk factors of pSS-RTA in order to carry out better screening and intervention. At present, studies have shown that many factors, such as ESR, are risk factors for the onset of pSS-RTA [20]. Some studies have also shown that the tissue biopsy of patients with pSS-RTA shows lymphocyte infiltration [21]. However, the distribution of peripheral blood lymphocytes subsets and cytokineins in patients with pSS-RTA has not been fully clarified. To further understand the immune cell status of pSS patients with or without RTA, we measured the level of lymphocytes subsets and cytokines in peripheral blood, and further explore their correlation.

We observed an increase in the secretion of various cytokines, including IL-2, IL-4, IL-6, IL-10, IL-17, IFN-γ and TNF-α in pSS-RTA patients, compared to pSS-no-RTA patients. This may be related to the activation of T cells. The pathogenesis of pSS complicated with RTA is not clear, which may be due to lymphocyte infiltration and immune complex deposition. Studies have shown that Th1 cytokines including IFN-γ、TNF-α and IL-2 are involved in cell-mediated inflammatory response, delayed hypersensitivity and activation of cytotoxic reaction[22]. Recent studies have shown that IL-2 can play different roles by activating different cells in the immune system [23, 24]. On the one hand, it acts as an inflammatory factor to activate effector T cells to promote immune response; on the other hand, it acts as an anti-inflammatory factor to induce Treg cell differentiation and inhibit Th17 cells to maintain immune tolerance, playing an important role in infection, autoimmune diseases and cancer [25]. In our study, compared with pSS-no-RTA patients, pSS-RTA patients have higher serum IL-2 levels, suggesting that high levels of IL-2 may promote immune response by activating effector T cells, thereby promoting the occurrence of pSS-RTA. In studies about systemic lupus erythematosus, IL-4 has been found to promote inflammatory responses in target organs, increase production of IgG and IgE, and is an essential inflammatory factor in the disease. High expression of IL-4 can be detected in the salivary glands of patients with pSS, particularly in patients with high levels of B-cell aggregation in the salivary glands [26]. IL-10 is mainly produced by monocytes and T lymphocytes and has an important immunostimulatory role as an important immunomodulator. IL-17 is a pro-inflammatory cytokine with a strong ability to recruit and activate neutrophils. It can induce inflammation and enhance inflammatory effects by inducing activation of T lymphocytes, stimulating macrophages, epithelial cells and fibroblasts to produce a variety of pro-inflammatory transmitters, while acting synergistically with various inflammatory cytokines such as TNF-α [27]. Study shows significantly higher peripheral blood IL-17 levels in pSS patients compared to healthy controls[28], and IL-17 expression was also found to be significantly higher in the salivary glands of pSS patients[29]. This study also found an increase in IgA in the pSS-RTA group, which was considered to be related to an overproduction of immunoglobulins due to the active function of B cells. We consider that the activated T cells produce more IL-4, IL-10, IFN-γ and TNF-α during pSS complicated by renal tubular acidosis, leading to the activation of B cells and thus more autoantibodies.

Treg cells play a critical role in suppression of excessive immune activation and maintaining immune homeostasis [30]. Conflicting findings regarding the effect of Treg cells in pSS immunopathogenesis [31–33]. Some researchers discovered that the percentage of Treg cells in pSS patients was reduced, and they came to the conclusion that decreased Treg could be responsible for the enhanced autoimmune response of effector cells [31, 34]. Meanwhile, other studies found that the Treg levels of pSS patients were either the same as or higher than healthy controls [10, 35]. The same as previous studies [32], our result also suggests that the percentage of CD4 + CD25 + Foxp3 + Treg cells have no significant differences in pSS and healthy controls. However, the absolute Treg cells count did decrease in pSS in comparison with health people. As a result, we hypothesize that the absolute number of Treg cells, rather than the percentage, was instrumental in the pathogenesis of pSS. There are various possible explanation for the reported discrepancies. As we all know, in regulatory T cells, the expression of CD25 is not necessary [36], while Foxp3 is the most representative marker of Treg. Moreover, CD25-FoxP3 + cells do not exhibit persistent suppressive activity [37]. The majority of current research focuses on CD4 + CD25 + Treg cells. However, in our study, CD4 + CD25 + Foxp3 + was used to represent Treg cells. Therefore, we speculate that one of the reasons for this difference is the lack of consistency between various monoclonal antibodies and analytical methods. Another explanation is that the baseline parameters of pSS patients included in various papers varied, such as age, gender, and comorbidities. These variables have a significant impact on the distribution of lymphocyte subsets. In our study, we compared Treg cells from patients with pSS-RTA and pSS-no-RTA to healthy controls, and the results showed that the absolute number of Treg cells was only reduced in pSS-RTA patients, but not in pSS-no-RTA patients. Furthermore, as compared to pSS-no-RTA patients, Treg cells in pSS-RTA patients reduced. As a result, we believe that the decrease of the absolute number of Treg cells may be an essential role in the complication of pSS with RTA, which may be a key characteristic of these patients. The deficiency of immune tolerance caused by the decrease of Treg cell level may be closely related to the pathogenesis of pSS-RTA. More studies are needed to determine the role of Treg cells in the pathophysiology of pSS-RTA. Th17 and Treg cells share a common precursor cell, an increase in Th17 cells is often together with a decrease in Treg cells. Imbalance of Th17 and Treg cells counts for various autoimmune diseases [38]. Same as previous studies, our findings indicate that the absolutely number of Th17 cells as well as the ratio of Th17/Treg was increased in pSS patients than HCs [39]. However, the number of Th17 cells and the ratio of Th17/Treg between pSS-RTA and pSS-no-RTA patients are not different from each other in statistic. It is the decrease in the absolute number of Treg cells that leads to insufficient immune tolerance, rather than the increase in Th17 cells, which may play a role in the pathogenesis of pSS-RTA. This study shows that the serum IL-2 level of pSS-RTA patients is significantly correlated with the Th17/Treg ratio, but it has no correlation with Treg cell level, suggesting that it is of great significance in monitoring Th17/Treg immune imbalance in patients.

Studies have shown that the decrease of NK cell number and functional characteristics in patients with autoimmune diseases is related to the progress of autoimmune process and the production of autoantibodies [40, 41], but it is unclear whether NK cell alterations are disease induced or pathogenetic. In addition, a temporal correlation between NK cell number or activity and disease progression in autoimmune diseases has been established, suggesting that NK cells may play an immunomodulatory role in disease pathology [42]. At the same time, NK cells are able to inhibit antibodies and thus play an important role in B-cell regulation [43]. IL-2 plays an important role in regulating the imbalance between autoimmunity and immune tolerance. Treg cells overexpress interleukin-2 receptor α(IL-2Rα),which has high affinity with IL-2. When IL-2 levels are low in the body, α-receptors preferentially bind IL-2, limiting the access of NK cells to IL-2 and thus inhibiting NK cells function[44]. However, when IL-2 levels are elevated in vivo and Treg cells are deficient, NK cells expressing IL-2Rβ and IL-2Rγ can respond to high levels of IL-2 by promoting their own proliferation and cytotoxicity, thereby increasing their self-response to target cells [45]. Studies have shown that NK cells suppress Treg cells by secreting INF-γ[46]. In our study, the levels of IL-2 and INF-γ in pSS-RTA patients were higher than those of pSS-no-RTA patients, while Treg cells decreased, suggesting that high levels of IL-2 promoted NK cell proliferation, while NK cells secreted INF-γ to inhibits the function of Treg cells. But our research also shows that the absolute number and proportion of peripheral blood NK cells was reduced in the pSS-RTA group compared to the pSS-no-RTA group, and the level of IL-2 was significantly negatively correlated with the number of NK cells. On the one hand, the decline in peripheral NK cells may be brought on by an increase in exocrine gland homing, which causes these glands to produce more Th1 cytokines and cytotoxic mediators, which in turn cause and perpetuate tissue inflammation [47]. On the other hand, disease processes and numerous bacterial and viral infections may be to blame for the excessive consumption of peripheral cells[47].

CD4 + T cells are a group of helper T cells that play an important regulatory role in the immune response. CD4 + T cells assist B cells to recognise thymus-dependent antigens and induce B cell differentiation and antibody production, while CD4 + T cells also assist cytotoxic T cells and macrophages in their immune functions. The T lymphocyte subpopulations work in concert or in constraint with each other to develop a modest immune response that allows them to clear antigenic foreign bodies without damaging their own tissues. In the peripheral blood of pSS-RTA patients, CD4 + T cells were decreased, making cellular immunity dysfunctional. On the one hand, the function of transmitting helper signals in the process of cellular immunity is decreased, thus failing to clear antigenic foreign bodies. On the other hand, the immune response is dysregulated, resulting in an imbalance between T lymphocyte subsets and an overproliferation of B cells that damage their own tissues. Activated CD4 + T cells are the main source of IL-2 production in vivo. According to Amado et al. [48] depletion of IL-2 did not affect Treg cells in mice, but did result in an increase in IL-2 production by CD4 + T cells. Moreover, in type 1 diabetes mice, when Treg cells are depleted, the level of IL-2 increases, but CD4 + T cells that secrete IL-2 do not increase [44]. This shows that when the number of Treg cells decreases, the IL-2 produced by CD4 + T cells increases in compensation[49]. This study found that the number of Treg cells and CD4 + T cells in peripheral blood of patients with pSS-RTA decreased, but the level of IL-2 increased, which may be due to the compensatory increase of the ability of CD4 + T cells to secrete IL-2.

The characteristics of Th2 cells within pSS pathogenesis have not been fully elucidated. In some surveys, Th2 cells were showed to be stimulate B cells to produce self-reactive IgE, which then contributes to the development of SS[50, 51]. Other studies have shown no significant role for Th2 cells in pSS. It is worth noting that the comorbidity of renal impairment was not examined individually for patients with pSS in the preceding investigation. Similarly, when all pSS patients, including pSS-RTA and pSS-no-RTA, were used in our analysis, there has not been a statistically significant decrease in the number of Th2 cells relative to the healthy controls. However, when we compared pSS-RTA and pSS-no-RTA patients alone with healthy controls, the numbers of Th2 cells were decreased in pSS-RTA, but not significant in pSS-no-RTA. Further, our study demonstrated that there were fewer Th2 cells in pSS-RTA than in pSS-no-RTA. As a result, we suggest that, to some extent, the decline in Th2 cells may be closely associated pSS in combination with RTA.

In order to exclude other possible influences on the immune cell status of pSS-RTA patients, logistic regression was used to exclude confounding factors. Multiple logistic regression suggested the increase of ESR, ALP as the risk factor for pSS-RTA and Treg as a protective factor for pSS-RTA. This shows that the amount of Treg cells has an independent relationship with the incidence of RTA in pSS, and that a reduction in Treg cells may play a significant role in pSS-RTA. Meanwhile, after adjusting for multiple confounders, the protective effect of Th2 cells and CD4 + T cell was eliminated and a further study could expand the sample size to further investigate the relationship between them and pSS-RTA. In line with our study, a multicentre study[20] also showed that the increase of ESR and ALP was independent risk factor for pSS-RTA. The study also found a significantly higher prevalence of fragility fractures in patients with pSS-RTA, suggesting that some patients with pSS-RTA may have abnormal bone metabolism and that clinicians should pay attention to screening for RTA in patients with pSS with elevated ALP, and also pay attention to the assessment of fracture risk in patients with pSS-RTA[20]. In conclusion, although the results of the multifactorial regression analysis cannot yet establish a clear causal relationship with the development of RTA in pSS patients, the decline in Treg cells offers a new way of thinking about the management of pSS-RTA.

This investigation still has several flaws. For starters, Due to the limited supply of renal biopsy tissues, this study focused on the changes of lymphocyte subsets in peripheral blood. Secondly, this is a retrospective study and the results of the multifactorial regression analysis have not been able to establish a clear causal relationship with the occurrence of RTA in patients with pSS and further validation in a prospective cohort study is needed in the future. In addition, more precise cytokine detection methods should be applied in future research to more accurately reflect the immune status of patients. At the same time, in this study, we observed the level of IL-2 in peripheral blood, without observe the activity of lymphocytes secreting IL-2. In the future study, we will further explore the source of IL-2 and the function of lymphocytes in the peripheral blood of patients with pSS-RTA, and further explore the pathogenesis of pSS-RTA.

Finally, our findings imply that the increase of ESR, ALP as the risk factor for pSS-RTA and Treg as a protective factor. In addition, the increase of serum IL-2 level and the decrease of peripheral blood NK cells may be involved in the pathogenesis of pSS-RTA, which provides a new understanding for the diagnosis and treatment of pSS-RTA.

**Table 1:** Characteristics of pSS-RTA group, pSS-no-RTA group and HCs

	pSS-RTA N = 25	pSS-no-RTA N = 54	HCs N = 33
Demographics			
Age (years)^b^	49.08 ± 11.59	50.28 ± 10.47	48.73 ± 12.62
Female, yes. n (%)	24(96.00)	53(98.15)	32(96.97)
BMI (kg/m2) ^b^	21.40 ± 1.56	21.80 ± 1.91	20.89 ± 1.85
Disease duration (months)^a^	59(27–147)	60(36–156)	-
Laboratory Characteristics			
WBC (*10^9/L) ^a^	4.39(3.70–6.87)	4.65(3.86–5.93)	6.08(4.88–7.40)*
Hb (g/L) ^b^	120.72 ± 16.19	122.02 ± 16.56	130.55 ± 9.40*
PLT (*10^9/L) ^a^	170.00(102.00-247.50)	201.50(151.75–253.00)	238(209.5-291.5)**
LY (*10^9/L) ^a^	1.15(0.91–1.52)	1.48(1.01-2.00)	1.98(1.65–2.37)***
ALP increased, n (%)	9(36.00)	6(11.11) **	0
BUN (mmol/L) ^a^	5.6(4.50–6.1)	4.6(3.70–5.70)	5.0(4.5–5.7)
Cr(umol/L) ^b^	71.07 ± 17.95	55.94 ± 15.98***	55.24 ± 7.10***
eGFR (ml/min*1.73m2) ^b^	88.16 ± 21.01	104.84 ± 11.77**	102.12 ± 7.70**
CO2-CP (mmol/L) ^a^	19.54(13.74–23.65)	25.0(23.7–25.50) **	25.40(23.05–26.35)**
Uric acid (umol/L) ^a^	294.00(233.00-349.00)	269.00(226.50-326.75)	260.00(220–292)
K (mmol/L) ^a^	3.16(2.53–3.43)	3.72(3.56–3.87) ***	3.97(3.76–4.17)***
Na (mmol/L) ^a^	140(137–142)	140(139–142)	141(139-141.5)
Cl (mmol/L) ^a^	110(107.25-114.75)	107(106–108) ***	105(103–106)***
Ca (mmol/L) ^a^	2.16(2.14–2.31)	2.23(2.17–2.31)	2.32(2.29–2.43)
Urinary pH ^a^	7.5(7.0-7.5)	6.5(6.0–7.0) ***	7.0(7.0-7.5)***
ESR increased, n (%)	22(88.00)	32(59.30) *	0
CRP increased, n (%)	4(16.00)	6(11.11)	0
IgG increased, n (%)	12/21(57.10)	21/50(42.00)	0
IgA increased, n (%)	11/22(50.00)	12/51(23.50) *	0
IgM increased, n (%)	1/21(4.80)	4/52(7.70)	0
C3 (g/L) ^a^	0.77(0.64–0.82)	0.78(0.70–1.08)	-
C4 (g/L) ^a^	0.16(0.11–0.21)	0.17(0.12–0.24)	-
Anti-SSA+/SSB+, n/N (%)	20/22(90.90)	44/49(89.90)	-
Anti-ENA+, n/N (%)	15/21(71.40)	20/39(51.30)	-
Anti-Ro52+, n/N (%)	16/21(76.20)	25/39(64.10)	-
Labial gland biopsies+, n/N (%)	7/12(58.33)	29/34(85.29)	-
ANA+, n/N (%)	20/22(90.90)	43/49(87.80)	-
RF increased, n/N (%)	16/22(72.7)	19/50(38.00) **	-
Clinical manifestations			
Dry mouth, n (%)	16(64.00)	39(72.22)	-
Dry eye, n (%)	21(84.00)	42(77.77)	-
Osteoporosis, n (%)	9(36.00)	9(16.67)	-
Nephrolithiasis, n (%)	6(26.08)	0	-

a Date with median and 25th and 75th percentiles

b Date with mean ± standard deviation

BMI: Body Mass Index; WBC: white blood cell; Hb: hemoglobin; PLT: platelet; LY: lymphocyte; ALT: alanine aminotransferase; BUN: blood urea nitrogen; Cr: serum creatinine; eGFR: estimated glomerular filtration rate; CO2-CP (mmol/L): carbon dioxide combining power; *P < 0.05, **P < 0.01, ***P < 0.001 vs. pSS-RTA

**Table 2:** Absolute counts and proportion of lymphocyte and CD4 + T cells in the peripheral blood in pSS and HCs

	All pSS patients N = 79	HCs N = 33	p-value
Numbers of lymphocytes (cells/µl)			
Total T cells	1047.98(730.89–1337.00)	1210.71(980.78-1337.47)	0.019 *
Total B cells	184.23(113.77-280.75)	175.81(127.66-235.72)	0.957
NK cells	120.51(80.68-176.55)	155.94(135.43-190.36)	0.004 **
CD4 + T cells	597.40(423.80-786.28)	647.50(550.17-712.18)	0.297
CD8 + T cells	440.23(297.62-572.06)	460.22(390.55-562.77)	0.123
Th1 cells	106.43(63.65-165.19)	133.95(55.71-176.68)	0.581
Th2 cells	6.07(4.11–7.87)	7.04(4.73–9.28)	0.209
Th17 cells	6.91(4.54–11.43)	5.68(3.67–8.27)	0.049 *
Treg cells	19.10(12.91–26.50)	23.04(19.38–28.20)	0.007 **
Proportions of lymphocytes (%)			
Total T cells	73.04(66.75–81.19)	74.23(73.49–80.96)	0.365
Total B cells	13.60(9.27–19.92)	10.96(8.23–14.04)	0.091
NK cells	8.75(6.16–11.84)	11.05(8.71–12.55)	0.018 *
CD4 + T cells	39.50(33.64–46.93)	39.78(36.03–44.59)	0.906
CD8 + T cells	31.16(24.63–39.65)	29.78(26.13–36.20)	0.786
Th1 cells	19.73(13.08–27.27)	19.51(12.24–25.58)	0.594
Th2 cells	1.12(0.78–1.53)	1.24(0.86–1.48)	0.934
Th17 cells	1.41(0.80–2.24)	0.89(0.62–1.25)	< 0.001***
Treg cells	3.66(2.82–4.62)	4.05(2.93–4.64)	0.174
CD4 + T/CD8 + T	1.35(0.91–1.90)	1.38(0.91–1.56)	0.542
Th1/Th2	17.08(10.69–25.70)	15.84(9.52–19.03)	0.473
Th17/Treg	0.41(0.23–0.68)	0.24(0.14–0.31)	0.001 **
Th1/Treg	5.73(3.30–8.54)	5.67(3.27–7.98)	0.461
Th2/Treg	0.31(0.22–0.44)	0.36(0.27–0.60)	0.449

Date with median and 25th and 75th percentiles

T: T lymphocyte; B: B lymphocyte; NK: natural killer cell; Th1: T-helper 1 cells; Th2: T-helper 2 cells; Th17: T-helper17 cells; Treg: regulatory T cells. *P < 0.05, **P < 0.01, ***P < 0.001

**Table 3:** Absolute counts and proportion of lymphocyte and CD4 + T cells in the peripheral blood in pSS-RTA, pSS-no-RTA and HCs

	pSS-RTA N = 25(A)	pSS-no-RTA N = 54(B)	HCs N = 33(C)	p-value	P, A vs. B	P, A vs. C	P, B vs. C
Total T cells	885.67 730.89-1061.91	1146.45 730.06-1473.90	1210.71 980.78-1337.47	0.007	0.176	0.005	0.267
Total B cells	181.46 132.59-299.48	186.60 104.24-245.16	175.81 127.66-235.72	0.764	-	-	-
NK cells	94.00 66.28–142.5	129.33 89.95-202.25	155.94 135.43-190.36	0.001	0.050	0.001	0.189
CD4 + T cells	505.77 413.51-638.52	617.86 462.42-808.13	645.00 542.23-712.18	0.028	0.048	0.046	1.00
CD8 + T cells	473.50 302.93-569.08	432.48 297.62-572.06	460.22 390.55-562.77	0.284	-	-	-
Th1 cells	88.69 45.64-165.19	109.24 73.96-163.21	133.95 55.71-176.68	0.541	-	-	-
Th2 cells	4.30 3.41–7.36	6.69 5.29–7.94	7.04 4.73–9.28	0.020	0.032	0.038	1.00
Th17 cells	6.13 4.18-10	9.21 4.65–12.18	5.68 3.67–8.27	0.033	0.259	1.000	0.044
Treg cells	15.75 7.79-20	21.37 15.99–29.91	23.04 19.38–28.20	0.000	0.002	0.000	0.560
Total T cells (%)	67.38 63.08–79.93	73.89 68.54–81.28	74.23 73.49–80.96	0.173	-	-	-
Total B cells (%)	14.58 9.82–26.04	13.16 9.27–18.29	10.96 8.23–14.04	0.764	-	-	-
NK cells (%)	7.00 4.51–10.86	9.31 6.94–13.4	11.05 8.71–12.55	0.008	0.135	0.006	0.383
CD4 + T cells (%)	36.05 31.92–40.6	40.91 37.04–47.99	39.78 36.03–44.59	0.075	-	-	-
CD8 + T cells (%)	35.09 26.51–43.73	30.23 23.85–38.02	29.78 26.13–36.20	0.280	-	-	-
Th1 cells (%)	18.56 14.72–29.62	19.18 13-25.63	19.51 12.24–25.58	0.781	-	-	-
Th2 cells (%)	1.07 0.74–1.46	1.13 0.84–1.53	1.24 0.86–1.48	0.921	-	-	-
Th17 cells (%)	1.43 0.78–2.28	1.41 0.87–2.18	0.89 0.62–1.25	0.002	1.000	0.025	0.003
Treg cells (%)	3.66 2.74–4.10	3.67 2.86–4.82	4.05 2.93–4.64	0.228	-	-	-
Th1/Th2	16.47 11.09–25.7	17.37 10.61–25.56	15.84 9.52–19.03	0.729	-	-	-
Th17/Treg	0.41 0.23–0.85	0.41 0.25–0.68	0.24 0.14–0.31	0.004	1.000	0.026	0.005

Date with median and 25th and 75th percentiles

T: T lymphocyte; B: B lymphocyte; NK: natural killer cell; Th1: T-helper 1 cells; Th2: T-helper 2 cells; Th17: T-helper17 cells; Treg: regulatory T cells. *P < 0.05, **P < 0.01, ***P < 0.001

**Table 4:** Univariate and multivariate logistic regression analyses for factors associated with the presence of RTA in pSS patients

Variables	Univariate		Multivariate	
	OR (95%CI)	P-value	OR (95%CI)	P-value
ESR increased	5.20(1.38–19.56)	0.015 *	17.07(1.09-267.31)	0.043 *
ALP increased	4.41(1.36–14.32)	0.014 *	12.89(1.50-110.57)	0.020 *
IgG increased	1.76(0.63–4.91)	0.282		
IgA increased	3.25(1.13–9.35)	0.029 *	1.08(0.21–5.44)	0.925
IgM increased	0.56(0.06–5.31)	0.613		
Positive RF	4.35(1.45–13.05)	0.009 **	1.20(0.24–5.99)	0.820
CD4 + T cells	1.00(0.99-1.00)	0.014 *	1.00(0.99–1.01)	0.773
Th2 cells	0.77(0.61–0.96)	0.020 *	0.78(0.55–1.11)	0.163
Treg cells	0.89(0.83–0.96)	0.002 **	0.90(0.81–0.99)	0.038 *
NK cells	0.994(0.988–1.001)	0.089		
NK cells %	0.942(0.860–1.032)	0.201		

Date with median and 25th and 75th percentiles

OR: odds ratio; 95%CI: 95% confidence interval; *P < 0.05, **P < 0.01

## Data Availability

The datasets used and/or analysed during the current study available from the corresponding author on reasonable request.

## Abbreviations

IL-2: Interleukin 2
IL-4: Interleukin 4
IL-6: Interleukin 6
IL-10: Interleukin 10
pSS: Primary Sjogren’s syndrome
RTA: Renal tubular acidosis
pSS-RTA: pSS complicated with RTA
pSS-no-RTA: pSS without RTA
TIN: Tubulointerstitial nephritis
Th1: T helper cell 1
Th2: T helper cell 2
Th17: T helper cell 17
Treg: Regulatory T
NK: Natural killer
IL-2R: Interleukin-2 receptor
CBA: Cytometric bead array
IQR: Interquartile rage
HCs: Healthy controls
ESR: Erythrocyte sedimentation rate
CRP: C-reactive protein
RF: Rheumatoid factor
ANA: Anti-nuclear antibodies
C3: Complement 3
C4: Complement 4
IgA: Immunoglobulin A
IgG: Immunoglobulin G
IgM: Immunoglobulin M
TNF-α: Tumor necrosis factor-α
IFN-γ: Interferon-γ
eGFR: Estimated glomerular filtration rate
ALP: Alkaline Phosphatase
BMI: Body Mass Index
WBC: White blood cell
Hb: Hemoglobin
PLT: Platelet
LY: Lymphocyte
BUN: Blood urea nitrogen
Cr: Serum creatinine
CO2-CP: Carbon dioxide combining power

## References

[1] Ramponi G, Folci M, Badalamenti S, Angelini C, Brunetta E (2020). Biomarkers and diagnostic testing for renal disease in Sjogren’s syndrome. Front Immunol.

[2] Chen X, Wu H, Wei W (2018). Advances in the diagnosis and treatment of Sjogren’s syndrome. Clin Rheumatol.

[3] Zhong H, Liu S, Wang Y, Xu D, Li M, Zhao Y (2022). Primary Sjogren’s syndrome is associated with increased risk of malignancies besides lymphoma: a systematic review and meta-analysis. Autoimmun Rev.

[4] Tak YJ, Kim JS, Lee KA, Kim HS, Jin SY (2022). Histological similarity between tubulointerstitial nephritis and salivary gland biopsy in primary Sjogren’s syndrome. Korean J Intern Med.

[5] Ramos-Casals M, Solans R, Rosas J, Camps MT, Gil A, Del Pino-Montes J (2008). Primary Sjögren syndrome in Spain: clinical and immunologic expression in 1010 patients. Med (Baltim).

[6] Yasutomo K (2003). Pathological lymphocyte activation by defective clearance of self-ligands in sytemic lupus erythematosus. Rheumatology.

[7] Zeher M (2010). Correlation of increased susceptibility to apoptosis of CD4 + T cells with lymphocyte activation and activity of disease in patients with primary Sjgren’s syndrome. Arthr Rhuem.

[8] Luo J, Ming B, Zhang C, Deng X, Li P, Wei Z et al. IL-2 Inhibition of Th17 Generation Rather Than Induction of Treg Cells Is Impaired in Primary Sjgren’s Syndrome Patients. other. 2018;9. 10.3389/fimmu.2018.01755 PMC6100298 30150979

[9] Liu MF, Lin LH, Weng CT, Weng MY (2008). Decreased CD4 + CD25 + bright T cells in peripheral blood of patients with primary Sjogren’s syndrome. Lupus.

[10] JE G, E FLKAJG (2005). CD4 CD25high regulatory T cells are not impaired in patients with primary Sjgren’s syndrome. J Autoimmun.

[11] Rusakiewicz S, Nocturne G, Lazure T, Semeraro M, Flament C, Caillat-Zucman S (2013). NCR3/NKp30 contributes to pathogenesis in primary Sjogren’s syndrome. Sci Transl Med.

[12] Izumi Y, Ida H, Huang M, Iwanaga N, Tanaka F, Aratake K (2006). Characterization of peripheral natural killer cells in primary Sjgren’s syndrome: impaired NK cell activity and low NK cell number. J Lab Clin Med.

[13] Agrawal S, Bharti V, Jain MN, Purkar PD, Verma A, Deshpande AK (2012). Sjogren’s syndrome presenting with hypokalemic periodic paralysis. J Assoc Physicians India.

[14] Roescher N, Vosters JL, Alsaleh G, Dreyfus P, Jacques S, Chiocchia G et al. Targeting the splicing of mRNA in autoimmune diseases: BAFF inhibition in Sjgren’s syndrome as a proof of concept. Mol Ther. 2014;22(4). 10.1038/mt.2013.275 PMC3982500 24304965

[15] Boyman O, Kolios AG, Raeber ME (2015). Modulation of T cell responses by IL-2 and IL-2 complexes. Clin Exp Rheumatol.

[16] Wu Y, Tian Z, Wei H (2017). Developmental and Functional Control of Natural Killer cells by cytokines. Front Immunol.

[17] Rosenberg SA (2012). Raising the bar: the curative potential of human cancer immunotherapy. Sci Transl Med.

[18] Vitali C, Bombardieri S, Jonsson R, Moutsopoulos HM, Alexander EL, Carsons SE (2002). Classification criteria for Sjögren’s syndrome: a revised version of the european criteria proposed by the american-european Consensus Group. Ann Rheum Dis.

[19] Shiboski CH, Shiboski SC, Seror R, Criswell LA, Labetoulle M, Lietman TM (2017). 2016 American College of Rheumatology/European League Against Rheumatism classification criteria for primary Sjogren’s syndrome: a consensus and data-driven methodology involving three international patient cohorts. Ann Rheum Dis.

[20] Zhang YY (2020). Clinical characteristics of patients with primary Sjogren’s syndrome Associated Renal tubular acidosis and neurological involvement.

[21] Moutsopoulos HM, Cledes J, Skopouli FN, Elisaf M, Youinou P (2010). Nephrocalcinosis in Sjgren’s syndrome: a late sequela of renal tubular acidosis. J Intern Med.

[22] Talaat RM, Elmaghraby AM, Barakat SS, El-Shahat M (2014). Alterations in immune cell subsets and their cytokine secretion profile in childhood idiopathic thrombocytopenic purpura (ITP). Clin Exp Immunol.

[23] Liao W, Lin JX, Leonard WJ (2013). Interleukin-2 at the crossroads of effector responses, tolerance, and immunotherapy. Immunity.

[24] Abbas AK, Trotta E, Marson DRS, Bluestone A. JA. Revisiting IL-2: Biology and therapeutic prospects. Sci Immunol. 2018;3(25). 10.1126/sciimmunol.aat1482 29980618

[25] Wu R, Li N, Zhao X, Ding T, Xue H, Gao C (2020). Low-dose Interleukin-2: Biology and therapeutic prospects in rheumatoid arthritis. Autoimmun Rev.

[26] Akiyama M, Yasuoka H, Yamaoka K, Suzuki K, Kaneko Y, Kondo H (2016). Enhanced IgG4 production by follicular helper 2 T cells and the involvement of follicular helper 1 T cells in the pathogenesis of IgG4-related disease. Arthritis Res Therapy.

[27] Kolls JK, Lindén A (2004). Interleukin-17 family members and inflammation. Immunity.

[28] Katsifis GE, Rekka S, Moutsopoulos NM, Pillemer S, Wahl SM (2009). Systemic and local interleukin-17 and linked cytokines associated with Sjögren’s syndrome immunopathogenesis. Am J Pathol.

[29] Sakai A, Sugawara Y, Kuroishi T, Sasano T, Sugawara S (2008). Identification of IL-18 and Th17 cells in salivary glands of patients with Sjögren’s syndrome, and amplification of IL-17-mediated secretion of inflammatory cytokines from salivary gland cells by IL-18. J Immunol.

[30] Ferreira LMR, Muller YD, Bluestone JA, Tang Q (2019). Next-generation regulatory T cell therapy. Nat Rev Drug Discov.

[31] Alunno A, Petrillo MG, Nocentini G, Bistoni O, Bartoloni E, Caterbi S (2013). Characterization of a new regulatory CD4 + T cell subset in primary Sjogren’s syndrome. Rheumatology (Oxford).

[32] Furuzawa-Carballeda J, Hernandez-Molina G, Lima G, Rivera-Vicencio Y, Ferez-Blando K, Llorente L (2013). Peripheral regulatory cells immunophenotyping in primary Sjogren’s syndrome: a cross-sectional study. Arthritis Res Ther.

[33] Chen L, Zhao, Guan, Lei Y et al. Elevated level of circulating CD4 + Helios + FoxP3 + cells in primary Sjogren’s syndrome patients. Mod Rheumatol. 2017. 10.1080/14397595.2016.1226470 27538522

[34] Banica L, Besliu A, Pistol G, Stavaru C, Ionescu R, Forsea AM (2009). Quantification and molecular characterization of regulatory T cells in connective tissue diseases. Autoimmunity.

[35] Sarigul M, Yazisiz V, Bassorgun C, Ulker M, Avci A, Erbasan F (2010). The numbers of Foxp3 + Treg cells are positively correlated with higher grade of infiltration at the salivary glands in primary Sjogren’s syndrome. Lupus.

[36] Fontenot JD, Gavin MA, Rudensky AY (2003). Foxp3 programs the development and function of CD4 + CD25 + regulatory T cells. Nat Immunol.

[37] Bonelli M, Savitskaya A, Steiner CW, Rath E, Smolen JS, Scheinecker C (2009). Phenotypic and functional analysis of CD4 + CD25- Foxp3 + T cells in patients with systemic lupus erythematosus. J Immunol.

[38] Lee GR. The balance of Th17 versus Treg cells in autoimmunity. Int J Mol Sci. 2018;19(3). 10.3390/ijms19030730 PMC5877591 29510522

[39] B OBJC AGMV (2018). Th17cells in primary Sjgren’s syndrome: pathogenicity and plasticity - ScienceDirect. J Autoimmun.

[40] Green MR, Kennell AS, Larche MJ, Seifert MH, Isenberg DA, Salaman MR (2005). Natural killer cell activity in families of patients with systemic lupus erythematosus: demonstration of a killing defect in patients. Clin Exp Immunol.

[41] Wither J, Cai YC, Lim S, McKenzie T, Roslin N, Claudio JO (2008). Reduced proportions of natural killer T cells are present in the relatives of lupus patients and are associated with autoimmunity. Arthritis Res Ther.

[42] Takahashi K, Miyake S, Kondo T, Terao K, Hatakenaka M, Hashimoto S (2001). Natural killer type 2 bias in remission of multiple sclerosis. J Clin Invest.

[43] James K, Ritchie AW (1984). Do natural killer cells regulate B-cell activity?. Immunol Today.

[44] Gasteiger G, Hemmers S, Firth MA, Le Floc’h A, Huse M, Sun JC (2013). IL-2-dependent tuning of NK cell sensitivity for target cells is controlled by regulatory T cells. J Exp Med.

[45] Levin AM, Bates DL, Ring AM, Krieg C, Lin JT, Su L (2012). Exploiting a natural conformational switch to engineer an interleukin-2 ‘superkine’. Nature.

[46] Oh KH, Lee C, Lee SW, Jeon SH, Park SH, Seong RH (2011). Activation of natural killer T cells inhibits the development of induced regulatory T cells via IFNγ. Biochem Biophys Res Commun.

[47] Shi L, Wang J, Guo HX, Han XL, Tang YP, Liu GY (2022). Circulating Th2 cell reduction and Th1/Th2 imbalance are correlated with primary Sjogren’s syndrome-associated interstitial lung disease. Arthritis Res Ther.

[48] Amado IF, Berges J, Luther RJ, Mailhé MP, Garcia S, Bandeira A (2013). IL-2 coordinates IL-2-producing and regulatory T cell interplay. J Exp Med.

[49] Li B, Guo Q, Wang Y, Su R, Gao C, Zhao J (2020). Increased serum Interleukin-2 levels are Associated with abnormal peripheral blood natural killer cell levels in patients with active rheumatoid arthritis. Mediators Inflamm.

[50] Pulendran B, Tang H, Manicassamy S (2010). Programming dendritic cells to induce TH2 and tolerogenic responses. Nat Immunol.

[51] Moriyama M, Hayashida JN, Toyoshima T, Ohyama Y, Shinozaki S, Tanaka A (2012). Cytokine/chemokine profiles contribute to understanding the pathogenesis and diagnosis of primary Sjgren’s syndrome. Clin Experimental Immunol.

